# Juvenile idiopathic arthritis in relation to perinatal and maternal characteristics: a case control study

**DOI:** 10.1186/s12969-017-0167-z

**Published:** 2017-05-11

**Authors:** Samantha W. Bell, Susan Shenoi, J. Lee Nelson, Parveen Bhatti, Beth A. Mueller

**Affiliations:** 1 Department of Epidemiology, School of Public Health, University of Washington, Seattle, WA USA; 2 Department of Pediatrics, Division of Rheumatology, Seattle Children’s Hospital, University of Washington School of Medicine, Seattle, WA USA; 3 Public Health Sciences Division, Fred Hutchison Cancer Research Center, Seattle, WA USA; 4 School of Medicine, University of California, San Francisco, San Francisco, CA USA; 5 University of California, San Francisco, 513 Parnassus Ave., Room S245, San Francisco, CA 94143 USA

**Keywords:** Juvenile arthritis, Maternal reproductive history, Case-control study, Perinatal factors, Birth certificate

## Abstract

**Background:**

Existing data on associations between maternal and early childhood exposures and juvenile idiopathic arthritis (JIA) risk is scant and inconsistent with previous studies showing potential role for prematurity, number of siblings and infections. We explored JIA and International League of Associations for Rheumatology (ILAR) JIA categories in relation to selected infant (birthweight, size-for-gestational-age, gestational age), and maternal (parity, delivery type, prior fetal loss) characteristics that may be markers for exposures related to two pathways (hygiene hypothesis, microchimerism) potentially associated with autoimmune disorder occurrence.

**Methods:**

A case–control analysis with 1,234 JIA cases and 5,993 birth year-matched controls was conducted. Exposure information was obtained from WA state birth certificates. Multivariable logistic regression was used to estimate adjusted odds ratios (OR) and 95% confidence intervals (CI) for associations with maternal and early life exposures for JIA and JIA categories.

**Results:**

Greater maternal parity was associated with a decreased OR for JIA (most marked for persistent oligoarticular JIA, OR 0.32, 95% CI 0.15; 0.71, p for trend = 0.0001). Prior fetal loss (except for oligoarticular JIA) was associated with an increased OR for JIA. Prematurity was associated with increased risk of enthesitis related arthritis (OR 1.9, 95% CI: 1.3–2.9) and rheumatoid factor positive polyarticular JIA (OR 2.2, 95% CI: 1.0–4.8).

**Conclusions:**

We observed associations of selected maternal factors with JIA, some of which varied across JIA categories. The findings of decreased ORs for JIA in relation to greater maternal parity may be consistent with the hygiene and microchimerism hypotheses. Future studies with biomarkers relevant to these hypotheses will help elucidate any associations.

## Background

Juvenile Idiopathic Arthritis (JIA) is a heterogeneous group of seven categories of chronic inflammatory arthritides in children with onset before 16 years of age [1]. JIA likely has a complex etiology, with multiple genetic and environmental factors contributing to its development [2]. Concordance rates of JIA among monozygotic twins are estimated at 25–40%; suggesting that environmental factors may influence JIA risk [3]. Evidence suggests that adult rheumatoid arthritis (RA) [4], JIA [5, 6], and other autoimmune diseases such as multiple sclerosis [7, 8] may be associated with maternal or early childhood exposures such as maternal parity, birthweight, or gestational age.

Two hypotheses have been suggested relating perinatal and maternal reproductive characteristics to JIA. The “Hygiene hypothesis” suggests that early exposure to infection results in decreased risk of childhood autoimmune disease [9] due to modulation of the developing immune system. Proxy measures often used to assess this include maternal parity (a greater number of prior births being consistent with a greater number of older daycare- or school-aged siblings at home and greater possibility of early infection); infant birthweight, small size-for-gestational age, or gestational age (small, or preterm infants may be less resistant to infection), and mode of delivery (vaginal deliveries may more likely elicit a normal immune response due to early exposure to vaginal flora). The microchimerism hypothesis relates to maternal and/or sibling origin microchimerism (harboring of cells or DNA from another genetically distinct individual) from exchange of cells/DNA *in utero* from the mother or older siblings during gestation [10]. Microchimerism may potentially increase or decrease disease risk based on the specificity of the acquired genetic material from another individual [11], and some studies suggest that its presence may alter rheumatoid arthritis (RA) risk in women [12–17] although we could locate no studies of this in JIA. It has been suggested that the decreased RA risk in parous women may be due to microchimerism among women with prior births [12]. Proxy measures potentially associated with increased microchimerism may include greater maternal parity (greater likelihood that infant has acquired genetic material from older sibling(s)); plurality (twins and higher-order multiples may have acquired genetic material from their sibling *in utero*); and a maternal history of prior fetal loss (maternally-retained genetic material from a fetus may be passed to subsequent offspring).

Lacking direct indicators of early prior infection relevant to the hygiene hypothesis, or of the presence of microchimerism, we explored associations of JIA and its individual categories in relation to selected maternal reproductive and infant characteristics as proxies for these, as assessed by birth certificate data among children with, and without JIA. Elucidation of these relationships will enhance understanding of possible JIA risk factors.

## Methods

### Study design

This case-control study identified JIA cases at a regional children’s hospital in the Seattle-Puget Sound region of Washington State using methods described previously [18]. Appropriate Institutional Review Board approvals were obtained. Potential JIA cases were identified though International Classification of Diseases (ICD-9), Ninth Revision diagnosis codes (720.0, 696.0, 714.32, 714.31, 714.30, 720.89, 714.33, 714.3) from 1997–2010 (*N* = 2,274) and linked to WA State birth records to identify children born in-state (*N* = 1,518). Controls were randomly selected in a 4:1 control: case ratio from remaining birth records, matched on year of birth (*N* = 6,072).

Chart review further refined JIA identification and categorized cases into ILAR categories including: polyarticular rheumatoid factor (RF) positive, polyarticular RF negative, oligoarticular arthritis (persistent and extended), psoriatic JIA, enthesitis related arthritis, systemic JIA, and undifferentiated JIA. We excluded 228 cases who did not meet ILAR criteria [1], and 38 cases with disease onset at > 16 years, resulting in 1,252 cases for analyses. After examining the OR for JIA in relation to plurality status, all remaining analyses were restricted to singleton infants only (1,234 cases; 5,993 controls) because characteristics (for example, birthweight and gestational age) of infants and pregnancies differ for singleton and multiple gestation pregnancies.

Exposure information from birth records included: birthweight (<2500, 2500- < 4000, 4000+ grams); size-for-gestational-age (SGA; small, AGA; appropriate, or LGA; large, based on above/below the 10th percentile for size/gestational age in Washington birth record standards); and gestational age (<37, 37- < 42, 42+ weeks). Maternal and infant factors relevant to the hygiene hypothesis included: number of prior births; delivery method (vaginal, cesarean (C) section), infant birthweight, gestational age and size for gestational age. To examine the microchimerism hypothesis, we examined birth order (as measured by maternal parity), plurality (singleton, twin/multiple gestation), and number of prior fetal losses (any; and by <20, 20+ weeks gestation).

### Statistical analysis

Multivariable logistic regression was used to estimate odds ratios (OR) and 95% confidence intervals (CIs) for associations, adjusted for maternal age, and the matching variable birth year. Other factors evaluated for possible effects on the relationships of interest included paternal age; maternal race/ethnicity, educational level, prenatal smoking, and marital status; medical insurance at birth (Medicaid vs. other); sex; and trimester prenatal care began. We adjusted for factors that altered the OR by greater than 10% in univariate analyses. Potential effect modifiers were evaluated by inspection of stratum-specific ORs and the Breslow-Day test for homogeneity. Trends were evaluated using Wilcoxon rank sum test. Analyses were initially conducted to examine JIA overall, and repeated for JIA categories (restricted to factors with at least 5 subjects in a cell). Analyses were conducted using Stata (version 11; StataCorp, College Station, Texas).

## Results

The majority of cases (67%) were female. Mothers of cases’ were more likely to be white (81%), married (80% v), college graduates (34%), have first trimester prenatal care (86%) and less likely to have Medicaid insurance (26%) (Table 1). Maternal gravidity was similar among cases and controls, with about 30% of mothers being nulliparous. Distribution of JIA categories included: oligoarticular; persistent (31.5%) and extended (5.9%), enthesitis related arthritis (21.2%), RF negative polyarticular (20.7%), systemic arthritis (5.8%), psoriatic arthritis (5.0%), RF positive polyarticular arthritis (4.9%), and undifferentiated arthritis (3.2%). The remaining 1.8% was polyarticular arthritis with unknown RF status.

**Table 1:** Infant and Maternal Characteristics of JIA Cases and non-JIA Controls born in Washington State 1997–2010

Characteristics	Cases (*N* = 1,252)^a^		Controls (*N* = 6,072)^a^	
	n	%	n	%
Infant Factors				
Female	843	67.3	2,903	47.8
Twin/multiple gestation^b^	18	1.4	79	1.3
Race/Ethnicity				
White	984	81.3	4,425	74.8
Black	37	3.1	316	5.3
Asian/Pacific Islander	60	4.9	426	7.2
Native American	38	3.1	150	2.5
Hispanic	91	7.5	598	10.1
Other	1	0.1	3	0.1
Maternal Factors				
Age (years)				
< 20	106	8.5	694	11.4
20–24	233	18.6	1,536	25.3
25–29	385	30.8	1,774	29.2
30–34	341	27.2	1,326	21.9
35+	187	14.9	739	12.2
Education (years)				
< 12	100	12.9	639	18.0
12	227	29.3	1,092	30.8
12–16	188	24.2	927	26.1
16+	261	33.6	888	25.0
Unmarried	250	20.0	1,577	26.1
Trimester of inception of prenatal care				
First	987	85.9	4,591	81.1
Second	133	11.6	897	15.8
Third	29	2.5	175	3.1
Medicaid insurance	282	26.3	1,789	37.0

^a^Numbers may not add up to totals due to missing data

^b^OR for association with JIA = 1.04, 95% CI: 0.62–1.74

### Infant and maternal characteristics in relation to JIA

Multiple gestation pregnancy was not associated with JIA (Table 1); OR 1.04, 95% CI: 0.62–1.74). Among singletons, birthweight >4000 g was associated with a decreased OR for JIA (OR 0.81, 95% CI: 0.67–0.98) (Table 2). The OR for JIA decreased with increasing number of prior maternal deliveries, with the lowest OR observed for children of women with two prior deliveries (0.68, 95% CI: 0.56–0.83 for 2 deliveries, p for trend =0.0007). No associations of JIA were observed with gestational age, size-for-gestational age, C-section delivery or maternal history of prior fetal losses.

**Table 2:** Infant and maternal characteristics among singleton Juvenile Idiopathic Arthritis (JIA) cases and controls born in WA State, 1997–2010

Characteristics	Cases (*N* = 1,234)		Controls (*N* = 5,993)			
	n	%	N	%	OR^a^	95% CI
Infant factors						
Gestational age (weeks)						
< 37	72	6.0	352	6.0	0.99	0.76, 1.29
37–42	1,030	85.5	4,903	83.9	1.00	reference
42+	102	8.5	587	10.1	0.88	0.70, 1.10
Size for gestational age						
SGA	102	9.4	496	9.6	1.01	0.81, 1.27
AGA	878	80.5	4,181	80.8	1.00	reference
LGA	110	10.1	500	9.6	1.01	0.81, 1.26
Birth weight (grams)						
< 2500	64	5.2	292	4.9	1.05	0.79, 1.39
2500–3999	1,026	83.3	4,880	81.7	1.00	reference
≥ 4000	142	11.5	803	13.4	0.81	0.67, 0.98
Maternal factors						
Cesarean Section						
Yes	256	20.7	1,110	18.5	1.08	0.93, 1.26
No	978	79.3	4,878	81.5	1.00	reference
Number of prior births^b^						
0	525	43.6	2,488	42.5	1.00	reference
1	418	34.7	1,893	32.3	0.95	0.82, 1.10
2	157	13.0	931	15.9	0.68	0.56, 0.83
3	60	5.0	325	5.6	0.71	0.53, 0.96
4+	44	3.7	216	3.7	0.72	0.51, 1.02
Any fetal loss (early or late)						
None	482	67.0	2,388	67.4	1.00	reference
1+	237	33.0	1,154	32.6	0.99	0.84, 1.18
Number of prior fetal losses (<20 weeks)^c^						
None	500	69.5	2,472	69.8	1.00	reference
1+	219	30.5	1,069	30.2	0.99	0.83, 1.18
Number of prior fetal losses (≥20 weeks)^c,d^						
None	698	97.1	3,429	97.1	1.00	reference
1+	21	2.9	104	2.9	1.09	0.67, 1.77

^a^Adjusted for maternal age and birth year

^b^p for trend ****P0.0007

^c^Restricted to 839 cases and 4,015 control whose mothers had a prior pregnancy

^d^Adjusted for maternal age, birth year, and paternal age

### Infant and maternal characteristics in relation to individual JIA categories

We observed an increased ORs for prematurity and RF+ polyarthritis (OR 2.23, 95% CI: 1.04–4.80) and enthesitis-related arthritis (OR 1.90, 95% CI: 1.27–2.86) (Table 3). SGA was associated with increased risk of undifferentiated JIA (OR 2.64, 95% CI: 1.18–5.94). Birthweight <2500 grams was associated with RF+ polyarthritis (OR 2.29, 95% CI: 1.03–5.13).

**Table 3:** Infant and maternal characteristics among singleton Juvenile Idiopathic Arthritis (JIA) case categories and controls born in WA State, 1997–2010

	Persistent Oligoarthritis		Extended Oligoarthritis		RF+ Polyarthritis		RF- Polyarthritis		Systemic JIA		Enthesitis Related Arthritis		Psoriatic Arthritis		Undifferentiated JIA	
*N* ^a^ *(%)*	*389*	*31.50%*	*73*	*5.90%*	*60*	*4.90%*	*256*	*20.70%*	*71*	*5.80%*	*262*	*21.20%*	*62*	*5.00%*	*39*	*3.20%*
	OR^b^	95% CI	OR^b^	95% CI	OR^b^	95% CI	OR^b^	95% CI	OR^b^	95% CI	OR^b^	95% CI	OR^b^	95% CI	OR^b^	95% CI
Infant factors																
Gest. age (wks)																
< 37	0.71	0.43, 1.18	-	-	*2.23*	*1.04, 4.80*	0.64	0.33,1.21	1.67	0.76, 3.69	*1.9*	*1.27, 2.86*	-	-	-	-
37–42	1	reference	1	reference	1	reference	1	reference	1	reference	1	reference	1	reference	1	reference
42+	0.88	0.60, 1.29	1.31	0.62, 2.75	0.74	0.29, 1.89	0.83	0.52, 1.33	1.07	0.48, 2.35	0.81	0.51, 1.29	0.79	0.31, 2.00	-	-
Size for gest age																
SGA	0.95	0.64, 1.40	0.79	0.31, 1.99	-	-	0.97	0.60, 1.55	1.03	0.44, 2.41	1.03	0.65, 1.62	1.31	0.55, 3.13	*2.64*	*1.18, 5.94*
AGA	1	reference	1	reference	1	reference	1	reference	1	reference	1	reference	1	reference	1	reference
LGA	1.16	0.82, 1.63	0.99	0.45, 2.18	1.42	0.60, 3.40	1.07	0.69, 1.65	0.8	0.32, 2.01	0.93	0.59, 1.47	-	-	-	-
Birth weight (g)																
< 2500	0.84	0.51, 1.40	0.24	0.03, 1.77	*2.29*	*1.03, 5.13*	1.1	0.63, 1.92	1.88	0.85, 4.15	1.18	0.69, 2.02	-	-	-	-
2500–3999	1	reference	1	reference	1	reference	1	reference	1	reference	1	reference	1	reference	1	reference
≥ 4000	0.85	0.62, 1.17	0.55	0.24, 1.28	0.66	0.26, 1.67	0.89	0.61, 1.31	-	-	0.96	0.67, 1.38	0.79	0.35, 1.74	-	-
Maternal Factors																
Cesarean Section																
Yes	1.17	0.92,1.66	0.93	0.52, 1.66	*1.8*	*1.00, 3.20*	1.07	0.78, 1.46	0.9	0.49, 1.65	1.14	0.84, 1.55	1.15	0.62, 2.13	0.84	0.35, 2.01
No	1	Reference	1	reference	1	reference	1	reference	1	reference	1	reference	1	reference	1	reference
No. prior birth^c,e^																
0	1	Reference	1	reference	1	reference	1	reference	1	reference	1	reference	1	reference	1	reference
1	0.88	0.69, 1.12	1.02	0.60, 1.74	0.72	0.38, 1.37	0.78	0.58, 1.06	0.92	0.53, 1.59	*1.36*	*1.02, 1.82*	1.15	0.65, 2.04	0.66	0.31, 1.41
2	*0.71*	*0.52, 0.98*	0.53	0.27, 1.04	0.67	0.27,1.67	*0.63*	*0.42, 0.95*	0.94	0.47, 1.86	0.83	0.56, 1.24	0.53	0.21, 1.33	0.6	0.25, 1.44
3	*0.55*	*0.32, 0.94*	-	-	1.77	0.75, 4.14	0.89	0.51, 1.53	0.64	0.24, 1.70	0.96	0.54, 1.69	0.71	0.26, 1.94	-	-
4+	*0.32*	*0.15, 0.71*	-	-	-	-	1.24	0.70, 2.18	-	-	0.65	0.31, 1.39	-	-	-	-
Any fetal loss																
None	1	reference	1	reference	1	reference	1	reference	1	reference	1	reference	1	reference	1	reference
1+	1.06	0.76, 1.47	0.77	0.35, 1.70	*2*	*1.00, 4.00*	*2.2*	*1.55, 3.12*	*1.9*	*1.00, 3.64*	*1.59*	*1.16, 2.17*	*2.85*	*1.57, 5.17*	1.42	0.61, 3.30
No. prior fetal losses (<20 w)^c^																
None	1	reference	1	reference	1	reference	1	reference	1	reference	1	reference	1	reference	1	reference
1+	1.11	0.80, 1.54	0.84	0.38, 1.88	1.71	0.83, 3.51	*2.08*	*1.46, 2.97*	1.68	0.86, 3.28	*1.56*	*1.13, 2.14*	*2.62*	*1.44, 4.78*	1.57	0.68, 3.65
No. prior fetal losses ((≥20 w)^c, d^																
None	1	reference	1	reference	1	reference	1	reference	1	reference	1	reference	1	reference	1	reference
1+	-	-	-	-	-	-	2.25	0.96, 5.25	-	-	1.9	0.86, 4.20	-	-	-	-

^a^Estimates do not include polyarthritis with unknown RF status

^b^All estimates are adjusted for maternal age and birth year

^c^Estimates are restricted to 839 case mothers and 4,015 control mothers with prior pregnancy

^d^In addition to maternal age and birth year, also adjusted for paternal age

^e^P for trends: persistent oligo: ****P0.0001; extended oligo, ***P0.0075. All others are > 0.05

Estimates in italics are statistically significant

The pattern we observed for overall JIA of decreasing ORs in relation to increasing number of maternal prior deliveries was marked for persistent oligoarthritis, with a monotonically decreasing trend ranging from 0.88 (95% CI: 0.69–1.12) for one prior birth, to 0.32 (95% CI: 0.15–0.71) for 4+ prior deliveries (p for trend =0.0001), and a suggestion of trend for extended oligoarthritis. Non-statistically significant decreased ORs were observed for greater numbers of prior deliveries for most other JIA categories, except for RF- (OR 1.24, 95% CI: 0.70–2.18 for 4+ prior deliveries) and RF+ (OR 1.77, 95% CI: 0.75–4.14 for 3+ prior deliveries) polyarthritis.

ORs were increased in relation to maternal fetal loss for psoriatic JIA (OR 2.85, 95% CI: 1.57–5.17); RF- polyarthritis (OR 2.20, 95% CI: 1.55–3.12); RF+ polyarthritis (OR 2.00, 95% CI: 1.00–4.00); systemic JIA (OR 1.90, 95% CI: 1.00–3.64) and enthesitis-related arthritis (OR 1.59, 95% CI: 1.16–2.17). Increased ORs were noted with maternal history of early fetal loss (<20 weeks) for enthesitis-related arthritis (OR 1.56, 95% CI: 1.13–2.14), RF- polyarthritis (OR 2.08, 95% CI: 1.46–2.96), and psoriatic (OR 2.62, 95% CI: 1.44–4.79).

## Discussion

We observed a decreased risk of JIA overall in relation to greater maternal parity, with a significant monotonic trend for persistent oligoarthritis, and suggestion of decreased ORs in relation to increasing maternal parity for most other JIA categories. This finding may be consistent with the hygiene hypotheses as children with older siblings likely have greater opportunity for infection exposure, both *in utero* and during post-natal life. Additionally a modest, non-statistically significant increased risk of JIA with Cesarean delivery similar to a Swedish registry report [5] provides weak support for the hygiene hypothesis, although this has not been supported in all studies [4].

Consistent with our data, a previous Australian clinic-based study showed that having any siblings (vs. none) was protective for JIA in (OR 0.46, 95% CI 0.28; 0.74, using clinic-based controls); a result slightly attenuated (OR 0.50, 95% CI 0.24; 1.02) when community controls were used [10]. An OR of 1.6 for JIA was observed in relation to being an only child in a Danish registry-based study [19]. In contrast, no risk reduction was observed in relation to having increased numbers of older siblings in a Swedish registry-based study [5]. Birth order was not found to be associated with JIA in two previous studies [6, 20].

The presence of microchimerism might increase or decrease JIA risk based on the nature of the acquired genetic material. An inverse association of JIA with greater maternal parity may be related to the presence of acquired genetic material carrying JIA-protective Human Leukocyte Antigen (HLA) alleles from an older sibling. In our study, most JIA category-specific risk estimates for maternal fetal loss were increased; a finding that could be consistent with the microchimerism hypothesis only if presence of increased levels of acquired microchimerism from a mother’s prior pregnancy carried JIA-associated risk HLA alleles [11]. However, it has been demonstrated that mothers of children with JIA have impaired reproductive capacity [21], so history of maternal fetal loss may also simply be an indicator of this observed association. Ultimately, we had no information about specific gene traits of children or their families, and this is speculation.

In contrast with previous studies from Sweden and United States [5, 6], high birthweight in our study was associated with a significantly decreased risk of JIA overall. Reasons for this difference are not clear.

Previous studies related to a gestational age effect vary. Although Swedish registry data suggest a borderline decreased OR in relation to preterm delivery [5], Shenoi et al [6] noted a significantly increased risk of JIA in relation to prematurity. While our results suggest no association with gestational age overall, enthesitis-related arthritis and RF+ polyarticular JIA had significantly increased ORs in relation to preterm gestation. Mothers of children with JIA have higher rates of preterm delivery [21] and the association of prematurity with JIA needs to be further explored in future studies.

The use of outpatient and inpatient medical records maintained by board-certified pediatric rheumatologists at a large regional children’s medical center, with data linkage to state birth records, are major strengths of this study enabling identification of a relatively large number of cases. Exposure information was assessed from birth records prior to onset and diagnosis of JIA, and was not subject to recall bias. Because of the known polygenic genetic predispositions and heterogeneity of the pathogenesis of differing JIA categories, it is important to examine these relationships separately for each category; to our knowledge, this is the first study to do so. Medical chart review allowed us to confirm and ascertain JIA categories, thereby reducing misclassification.

Some limitations of our study include possible inaccuracies in reporting of mother’s reproductive history; in particular, inaccurate reporting of fetal loss on the birth certificates may have lead to non-differential misclassification, biasing the OR estimates towards the null. Birthweight and number of prior live births, however, have been assessed as reliable in comparison to medical records [22]. There is potential for unmeasured confounding due to unrecognized pregnancy loss, psychosocial support factors, and lack of information on fertility, or residual confounding due to the necessity of reliance on birth records for exposure information. The inability to measure these could lead to residual confounding in our risk estimates and could bias the estimates either way. Although we attempted to identify all children with JIA at the sole facility with board-certified pediatric rheumatologists in Washington State during the study period, and free care is available for families without resources, it is possible that our JIA cases may not be representative of all children with JIA in Washington State. Some cases may have been treated by adult rheumatologists or other practitioners outside this facility, or they may differ from the underlying population in ways that we could not measure. We attempted to evaluate any related bias due to this, although adjustment for socio-economic and other factors potentially related to care access had no effect on the results. Our inability to examine the presence of younger siblings is also a limitation.

## Conclusions

We observed decreased JIA risk with increasing parity, which potentially supports the hygiene or microchimerism hypotheses, with consistent results demonstrated in many JIA categories. Of note, our results indicate the need to consider JIA categories as separate entities; indeed our results were often inconsistent across categories, suggesting potentially different etiologies. Given the current lack of preventative or curative treatments for JIA, further studies, particularly those capable of examining category-specific JIA associations, are warranted. A better understanding of these associations will help inform prevention programs and hopefully reduce the burden of this debilitating disease.

## Abbreviations

CI: Confidence interval
ICD-9: International classification of diseases
JIA: Juvenile idiopathic arthritis
OR: odds ratio
RF: Rheumatoid factor
